# A state-of-the-art examination of disaster management in Sierra Leone: the implementation drawbacks, research gaps, advances, and prospects

**DOI:** 10.1186/s40677-022-00224-3

**Published:** 2022-11-03

**Authors:** Bashiru Turay, Sheku Gbetuwa

**Affiliations:** 1 Department of Geography, University of Bonn, Bonn, Germany; 2 Institute for Environment and Human Security, United Nations University (UNU-EHS), Bonn, Germany; 3 Institute of Geography and Development Studies, School of Environmental Sciences, Njala University, Moyamba, Sierra Leone; 4 Department of Spatial Planning, Technical University Dortmund, Dortmund, Germany

**Keywords:** Disaster management, Government, Sierra Leone, Climate change, Risks, Hazards

## Abstract

Unfolding events have shown that Sierra Leone is enduring various disasters at a worrying rate. While progress is being made in disaster management, activities that degrade the fragile ecosystem, exacerbated by climate change, poverty, and bad governance, remain growing concerns. Amid these concerns, there is inadequate information to take appropriate actions. The authors wish to provide a solution by examining 35 publications from various scholarly and grey literature and raw data sources, following a critical review process designed to expose the implementation drawbacks, research gaps, advances, and prospects in disaster management in Sierra Leone. The study results show that lapses in data management, fewer professionals, and inefficiencies in communication are the most pressing considerations for improving disaster management in the country. Inadequate funds to implement management plans remain pervasive. Following these findings, we recommend that all first responders be routinely exposed to international tabletop exercises and simulated disaster response training to help them build their capacities and learn from other countries. Students in the relevant disciplines should be encouraged to participate in these exercises to facilitate early learning. Also, management initiatives should consider gender equity, the situations of the disabled, and other vulnerable groups at all levels of planning and implementation of activities. Future studies should assess the influence of social media on disaster management research and practice in the country.

## Introduction

Sierra Leone has experienced several disasters such as landslides, civil war, Ebola Virus Disease and frequent flooding. Most of these were either caused or exacerbated by human influences and have resulted in injuries, deaths, property losses and damages, and the interruption of daily life support services, all of which have seriously affected the country's growth and development (DMD and ONS 2006).

Previous literature has pointed out that environmental degradation, climate change, poverty, and bad governance mainly contribute to disasters in the country (Clark-ginsberg 2014). These factors ultimately combine to make Sierra Leone's disaster management more reactive and focused on responding rather than being proactive and focused on avoiding, preventing and mitigating disaster impacts (Miles et al. 2021)*.*

Another obstacle to disaster management is the politicisation of national issues. People spread fake news and baseless rumours to gain political advantage during public catastrophes (Sawaneh 2020). This act, added to the state officials' contradictory actions during disasters and emergencies, leaves questions and doubts in the victims' minds about their fate during such circumstances (Sesay and Bradley 2022).

Additional problems that have been identified to hinder disaster management are conflicting mandates among institutions with a role in disaster management. For instance, state institutions such as the National Protected Area Authority and the Forestry Department are mandated to preserve Freetown's hills as natural assets. At the same time, the Ministry of Lands is sometimes interested in awarding such lands for estate development as part of its land protection, planning and allocation mandates. This conflict in institutional mandate can sometimes affect land conservation and other disaster mitigation interventions. In all these, individuals and communities already marginalized bear the brunt of the consequences (Sesay and Bradley 2022).

A "disaster" in this context implies a significant disruption of the country's wellbeing, safety, and functioning at any spatial level brought on by dangerous events interacting with insecurity, capacity, and exposure, leading to losses in lives, property, and environmental resources that may require external humanitarian assistance.

This study adopts the UN description of disaster management, which is the organization, planning, and implementation of measures for the prevention and mitigation, preparedness, response, rehabilitation/reconstruction/recovery from a disaster (UNDRR 2022).

The terms "emergency management" and "disaster management" are sometimes used interchangeably. While there is some overlap, an emergency refers to hazardous situations that do not cause a significant interruption in a community's or society's functioning (UNDRR 2022).

Researchers and practitioners have made efforts to improve disaster management in the country by doing relevant assessments. For instance, the then Ministry of Lands, Country Planning and the Environment and the Freetown City Council evaluated natural disasters and risks in Freetown to improve the city's disaster mitigation (MLCPE and FCC 2014). Likewise, a national risk map (Groen and Jacobs 2012), disaster preparedness baseline (Pacific Disaster Center 2020), etc., have been developed. Researchers have investigated different risks, hazards, and disasters (Kamara et al. 2022; Morton Hamer et al. 2019; Osuteye et al. 2017; Shin et al. 2018)and their effects on the long-term economic sustainability of the country (Dumbuya and Nirupama 2017).

However, there are existing gaps in that most of the previous research either do not give a detailed national view, missed recent happenings, or failed to capture the updated context of national disaster management covered in The National Disaster Management Agency Act of 2020 (2020). The aforementioned issues have resulted in inadequate information for effective risk management in the country, making room for disaster mismanagement (Government of Sierra Leone 2021).

Regarding the above and the stride to provide contemporary information and implementable recommendations for improvement, this paper, therefore, assess the state of disaster management in Sierra Leone through a critical review of relevant documents about disaster and their management in the country.

As such, the objective of this study is to examine disaster management in Sierra Leone with a focus on exposing the implementation drawbacks, research gaps, advances and prospects.

“The results and discussion” section of this paper present the analysis of disasters and their management, implementation drawbacks, research gaps, and the presentation of the advances and prospects. Lastly, presented are the conclusion and recommendations that detail measures that, if implemented, will provide a solution to disaster management problems in the country.

## Methodology

This study utilized scholarly, grey literature and raw data drawn from various sources, following a systematic review process. Scholarly articles were obtained from Scopus and Google Scholar. Raw data were obtained from the international disasters database website at https://public.emdat.be/data, and the Directorate of Health Security and Emergencies, Ministry of Health and Sanitation, Sierra Leone website http://dhse.gov.sl/. Grey literature was obtained from the United Nations Framework Convention on Climate Change https://www4.unfccc.int/sites/NAPC/Pages/national-adaptation-plans.aspx; the International Federation of Red Cross and Red Crescent https://www.ifrc.org/docs/idrl/671EN.pdf, https://disasterlaw.ifrc.org/media/3020; World Health Organization https://www.afro.who.int/publications/sierra-leone-national-action-plan-health-security-2018-2022; Reliefweb https://reliefweb.int/disaster/fr-2021-000169-sle; Sierra Leone Urban Research Centre https://www.slurc.org/; Save the children https://www.savethechildren.org.uk/blogs/2017/beating-pneumonia-sierra-leone; Awoko www.awokonews.sl and the patriotic vanguard https://www.thepatrioticvanguard.com/on-epidemics-and-pandemics-in-sierra-leone.

The authors had to include the grey literature mentioned above to adjust for the insufficient relevant scholarly literature and to capture recent happenings not yet found in scholarly articles. The results from different sources were complementary and aided in catching a holistic view of the subject matter and reducing data bias.

### Review procedure

#### Criteria for inclusion

Criteria for including work in this review is that it should be a publication about managing any disaster at any spatial level in the country. Alternatively, it should be an institutional policy or act for national disaster management. These criteria were set to focus only on national disasters and their management.

#### Raw data search

On the international disaster database website, all the disaster classifications (Geophysical, Meteorological, Hydrological, Climatological, Biological, Extra-terrestrial, Technological, and Complex Disasters) in the platform were checked and applied to Sierra Leone, covering all the disaster years of records in the database from 1900 to 2022. The total number of lab-confirmed cases and deaths due to the Corona Virus disease in Sierra Leone was taken from the Directorate of Health Security and Emergencies, Ministry of Health and Sanitation website.

#### Literature search and screening

The following Scopus database search string "TITLE-ABS-KEY (disaster AND management AND in AND sierra AND leone)" yielded 39 results. The Scopus database search was complemented with a literature search in google scholar using the search string "Incident crisis emergency catastrophe disaster management in Sierra Leone". The Google Scholar search yielded 18,400 results. The authors sorted the result by relevance and had 36 publications consistent with the research topic drawn from the first 15 web pages.

The retained results from Google Scholar (36) and Scopus (39) were combined and checked for duplication. This process left a total of 52 results. The titles and abstracts of the documents were screened for their consistency with the research objective. We retained 30 publications after this process and had their full texts read. Five (5) more results were obtained using a direct search of the relevant institutional websites referenced in the literature. The purpose was to capture updated, not recorded, or missing disasters and impact records.

The final sample contains thirty-five publications, which include nineteen journal articles, eight reports, four government policies and acts, and four news/blogs. The authors examined them under the following themes: disasters and their management; implementation drawbacks; authors' recommendations; research gaps; advances and prospects.

## Results and discussion

### Disasters and their management

**Fig. 1:**
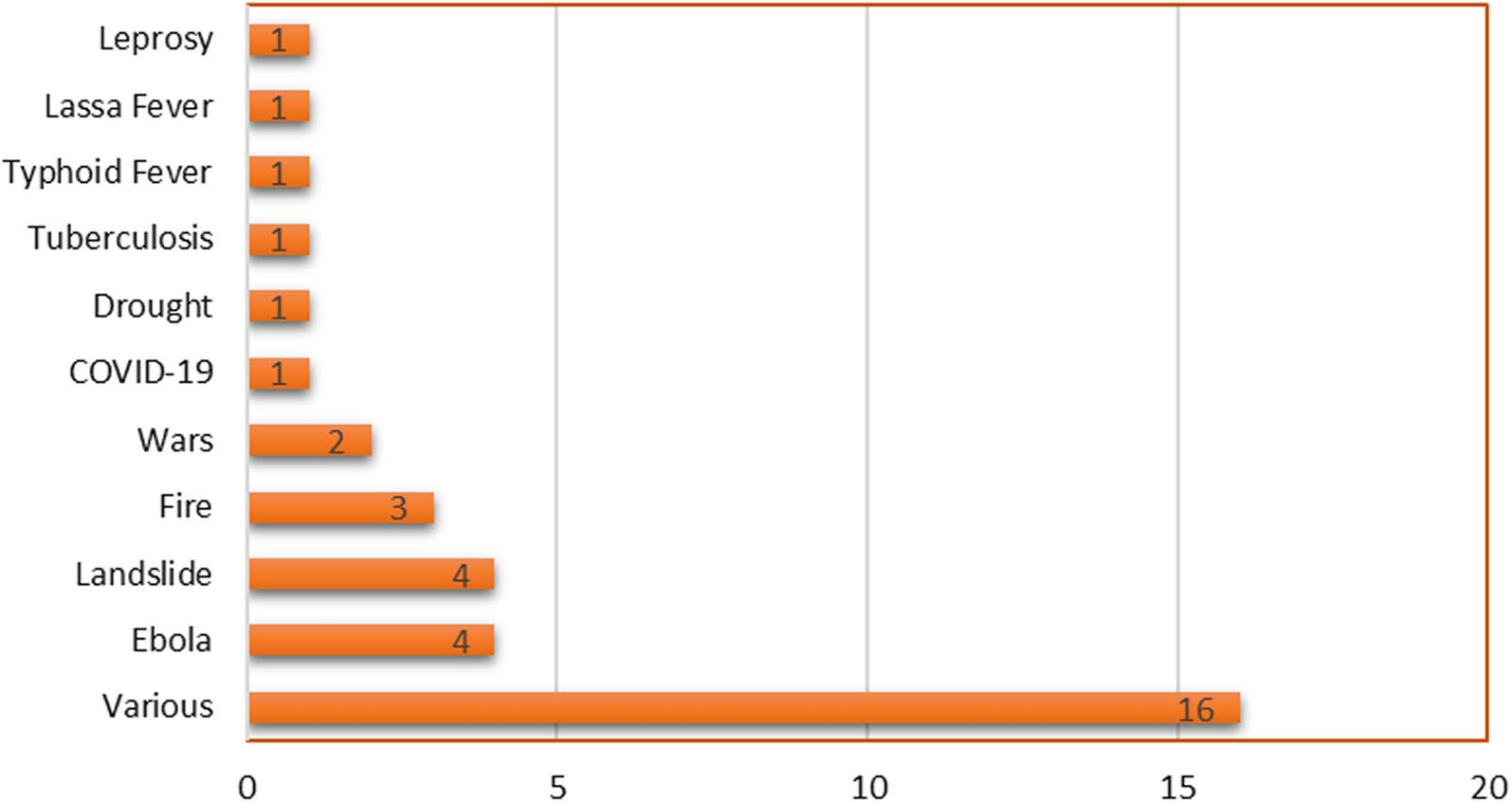
Disasters investigated by studies on this topic as drawn from the sample. *Source* Authors

The authors examined the disasters, periods of occurrence, impacts and their management. A total of 16 different disasters were found in the literature. The review shows that Ebola Virus Disease (n = 4) and Landslide (n = 4) are the disasters most independently researched in Sierra Leone, followed by Fire, Wars, COVID-19, Drought, Tuberculosis, Typhoid Fever, and Lassa Fever (See Fig. 1).

Table 1 summarises disasters, periods of occurrence, impacts, and sources.

**Table 1:** A breakdown of disasters in Sierra Leone, period of incidence, impacts, and sources

Disaster	Period of incidence	Cases-impacts	Sources
Cholera	1998–2013	Confirmed 31,653 cases and 589 deaths	Government of Sierra Leone (2019)
Civil war	1991–2002	Estimated 70,000 casualties and 2.6 million displaced people	United Nations Development Programme (2006)
COVID-19	2020-Date	7681 confirmed cases and 125 deaths	Directorate of Health Security and Emergencies (2022)
Dysentery	1999–2000	3094 total affected and 132 deaths	EM-DAT (2022)
Ebola virus disease	May 2014–March 2016	8706 infected cases and 3956 deaths	Government of Sierra Leone (2019), Miles et al. (2021)
Fire	2021	123 seriously injured, and at least 101 people dead. Between 189 and 200 structures destroyed, 7093 people affected	IIED (2021, Relief web (2021)
Flooding	2005, 2007, 2009, 2010, 2011, 2015 and 2019	55,888 total affected and 161 deaths	Clark-ginsberg (2014), EM-DAT (2022)
Human immunodeficiency virus (HIV)	1984—date/ongoing	An estimated 80,000 Adults and children living with HIV	National Disaster Management Agency Act (2020)
Landslide	9th August 2010 and 17th August 2017	16 deaths and 5 affected; 1118 deaths at least, 11,821 total affected	EM-DAT (2022), Miles et al. (2021)
Lassa fever	Since 1970s	782 lab confirmed cases between 2007 and 2017	Government of Sierra Leone (2019), Shaffer et al. (2021)
Leprosy	N/A	133–140 cases in 2015 and 2016; 446 in 2008	Awoko (2018)
Malaria	N/A	1649,644 confirmed cases; 2257 deaths in 2017	Government of Sierra Leone (2019)
Severe pneumonia	N/A	114,127 confirmed case and 918 deaths in 2016 and 2017	Government of Sierra Leone (2019)
Smallpox	1905; 1915–16, 1933, 1946, 1955–56, 1957, 1962 and 1967–68	Approximately 7800 affected	Cummings et al. (1971), The Patriotic Vanguard (2014)
Tuberculosis (TB)	Ongoing	An estimated incidence of 295 cases per 100,000 population. 2164 deaths between 2014 and 2016	Kamara et al. (2022)
Yellow fever	1815–1884	1041 fatalities	The Patriotic Vanguard (2014)

The country has suffered a series of cholera outbreaks between 1998 and 2013. These outbreaks have caused the loss of more than 589 lives. After 2013, cholera has still been infecting communities but not at an epidemic level. The disease is connected to the poor sanitation conditions of many communities in the country. The Ministry of Health and Sanitation and its partners, assisted victims during the outbreaks and helped prevent the spread of the disease through treatments and a series of sensitization campaigns run by local community disaster management (CDMCs) and water, hygiene, and sanitation (WASH) committees (Clark-ginsberg 2014; Government of Sierra Leone 2019).

The civil war, which lasted for 11 years, from 1991 to 2002, was one of the country's worst disasters and humanitarian crises. The government of Sierra Leone, with support from its international partners, ended the civil war in 2002 after an 11 years of brutal killings and destructions of homes and properties. The United Nations is among the key players that helped to end the war in the country. The focus of ending the war was on demobilization, disarmament and reintegration, and return and resettlement of displaced people (United Nations Development Programme 2006).

The ongoing COVID-19 pandemic, a respiratory infectious disease, was first recorded in Sierra Leone on March 31st, 2020. Through May 31st, 2022, the country has recorded 7682 cases and 125 deaths. Results show that the implementation of public health measures and the vaccination of people have reduced the fatality of the disease, and people are gradually returning to their normal livelihoods (Directorate of Health Security and Emergencies 2022).

An outbreak of dysentery was confirmed in November 1999 in Sierra Leone from samples obtained from the Western Area. By January 6th, 2000, the outbreak caused by Shigella flexneri and Shigella dysenteriae type 1 (Sd1) had reached Moyamba and Koinadugu districts and caused a total of 132 deaths and 3094 cases (EM-DAT 2022; MLCPE and FCC 2014).

The Ebola virus disease (EVD), one of the deadliest disasters in the country, struck with a record 3956 deaths. The country reported its first case on May 24, 2014. The government of Sierra Leone and its partners utilized rapid response teams and strong community involvement to control the disease transmission. The measures, together with the Ebola vaccination, proved active in the fight against the disease. The EVD ended on November 7th, 2015 (Government of Sierra Leone 2019; Miles et al. 2021).

Fire-related disasters are another disaster that has been occurring frequently in the country. On the 5th of November, 2021, a fuel tanker exploded in Wellington, Freetown, and resulted in at least 101 deaths and 123 seriously injured people. This fire event led to the activation of the public health emergency management and operation centre. Medical practitioners responded to provide emergency medical care to the victims at the various health centres they were in (IFRC 2021). Another fire incident was at Susan's Bay, which was estimated to have destroyed over 200 houses and made more than 1000 residents homeless (IIED 2021).

Flooding is a frequent occurrence in Sierra Leone, with potential occurrences in every year's rainy season, causing the loss of lives and properties and serious economic impacts. Flooding causes cannot be unconnected to the dumping of waste in drainages, improper drainage infrastructure, unplanned buildings, etc. The NDMA and its partners are working on a solution to mitigate the effects of flooding to reduce its impacts (Clark-ginsberg 2014; EM-DAT 2022).

The country is experiencing a varied and widespread HIV epidemic. According to the demographic and health survey, HIV prevalence is 1.7% among women and men aged 15–49 in Sierra Leone. HIV prevalence is greater among women than men (2.2% vs 1.1%). The prevalence of HIV is higher in cities than in rural areas (2.3% vs 1.2%). To minimize HIV-related morbidity and mortality across the country, the government of Sierra Leone and its partners are striving to restrict the transmission of HIV and AIDS through public awareness campaigns and the provision of free antiretroviral and other drugs to patients (Government of Sierra Leone 2019; Statistics Sierra Leone 2019).

Significant landslide and mudflow occurrences occurred in and around Sierra Leone's capital city of Freetown on August 14, 2017. Following 3 days of severe rain, hundreds of structures in the city were damaged or destroyed by mud and debris, killing 1141 people and displacing over 3000 people. The landslides were caused by the region's unique geography and climate—with Freetown's elevation close to sea level—and the region's generally weak infrastructure, and the loss of protective natural drainage systems due to deforestation. Another landslide struck the western area in August 10 2010, but it had fewer consequences than the incident on 17 August 2017 (EM-DAT 2022; Miles et al. 2021).

Sierra Leone has a significant prevalence of Lassa fever disease, a zoonotic viral hemorrhagic disease, notably in its Eastern Province, where 782 lab-confirmed cases were reported between 2007 and 2017. Lassa fever disease is transmitted to humans through the contamination of broken skin or mucous membranes by direct or indirect contact with infected rodent excreta on floors, home surfaces, and food or water. The country has an isolation and treatment centre in Kenema Government Hospital (Government of Sierra Leone 2019; Shaffer et al. 2021).

Leprosy is an infectious disease that causes disfiguring skin sores and nerve damage in the arms, legs, and other body parts. The disease has been in existence since ancient times. It is generally associated with scary negative perceptions and stories of leprosy victims being shunned as outcasts. Every year, new cases of leprosy emerge in Sierra Leone. In 2015 and 2016, 133–140 instances were discovered, compared to 446 in 2008 (Awoko 2018).

Sierra Leone has one of the world's highest malaria burdens. The disease threatens the whole population of Sierra Leone, and it is one of the country's top causes of mortality and illness. Between 2010 and 2015, malaria deaths in Sierra Leone decreased dramatically. This decrease is connected to various efforts, including enhanced diagnostic test availability, free treatments, and the widespread distribution of insecticide-treated nets (Clark-ginsberg 2014; Government of Sierra Leone 2019).

The government of Sierra Leone referred to pneumonia as the most common cause of under-five mortality. There were 114,127 confirmed cases and 918 deaths in 2016 and 2017. The disease is slowly being put under control with the administration of pneumococcal vaccines (PCVs) and antibiotics (Government of Sierra Leone 2019; Save the Children 2017).

Smallpox epidemics appear to have been more common in historical Sierra Leone than any other disease. Many people rejected the smallpox vaccine due to having no experience with it, while others did not take it for fear that it was an act of fetish and sorcery. The disease was finally eradicated in Sierra Leone in 1969 (The Patriotic Vanguard 2014).

Sierra Leone is high-burden tuberculosis (TB) country, ranking among the top 30 countries with the disease. Sierra Leone's government and partners are working to improve TB diagnosis, surveillance, and treatment in all parts of the country, as well as raise awareness of available treatments. Treatment for tuberculosis has been offered free in 170 locations across the country (Government of Sierra Leone 2019; Kamara et al. 2022).

Yellow fever epidemics were common throughout the nineteenth century. The first case of yellow fever in this region was discovered in 1764. Since the introduction of the vaccines in the 2000s, the number of deaths from the disease has decreased (The Patriotic Vanguard 2014).

### National disaster management framework

The National Disaster Management Agency was established in Sierra Leone in 2020 to manage disasters and similar emergencies throughout the country and to develop community capacity to respond effectively to disasters and emergencies (National Disaster Management Agency Act 2020).

Disasters in Sierra Leone, according to the literature, can be referred to, and dealt with based on three levels; one, two, and three (DMD and ONS 2006). Minor disasters within the local government's, community's, and stakeholders' reaction capabilities and result in only a modest requirement for national help are classified as Level One. A disaster that would likely exceed local capabilities and necessitate a wide spectrum of national help is classified as Level Two. Level three denotes a major disaster requiring massive national assistance, including military intervention and or international assistance (DMD and ONS 2006).

Under provision 29 of Act Number 6 of the Sierra Leonean Constitution of 1991, the President can declare a state of emergency for a disaster of national concern. The National Platform is in charge of informing the President about the impending disaster. When the President declares a state of emergency in a region, the resident minister in charge issues or authorizes the issuance of instructions for that region, and the district council chairmen and paramount chiefs can then take the lead at their respective administrative levels (National Disaster Management Agency Act 2020).

**Fig. 2:**
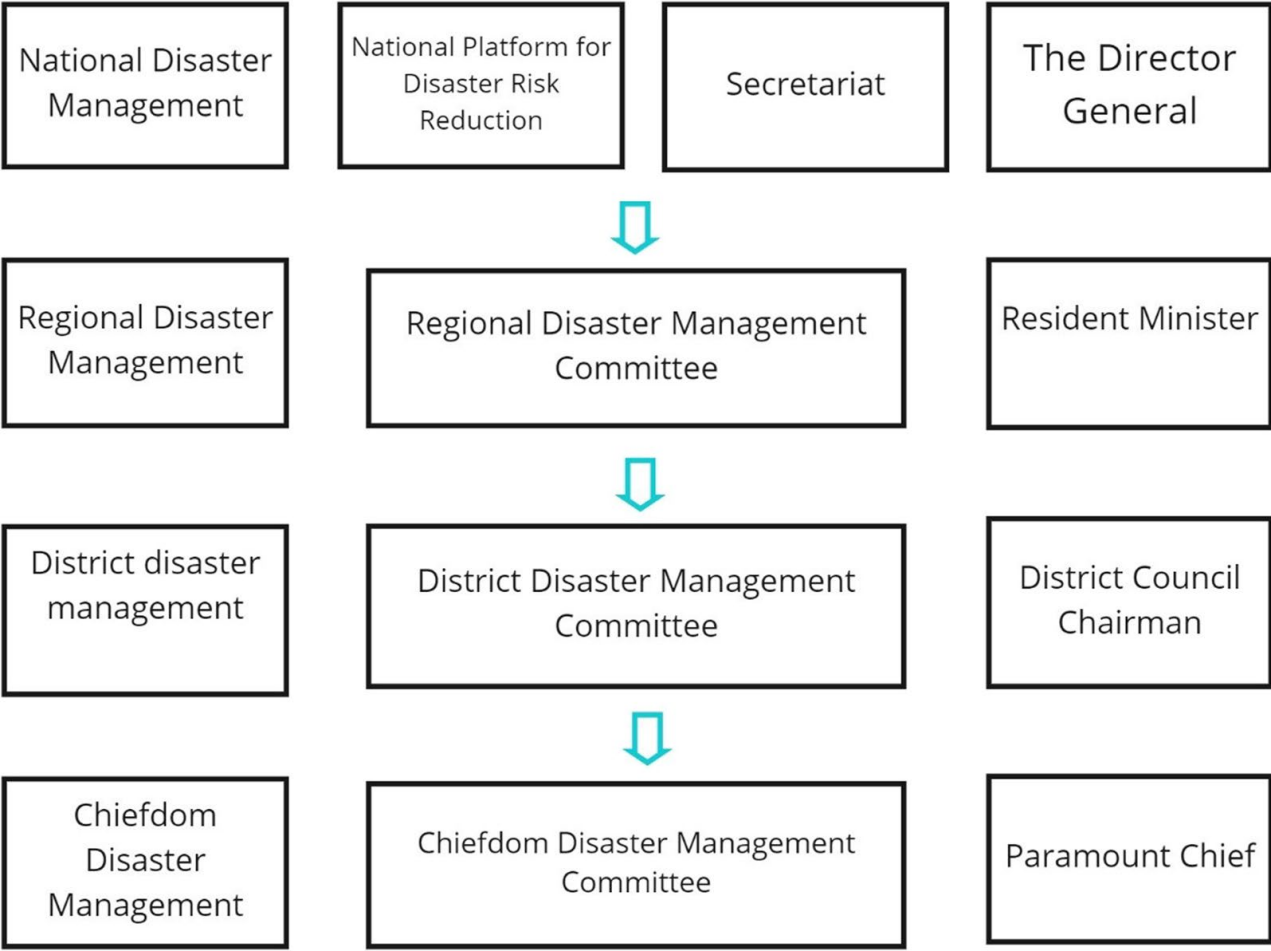
The national disaster management agency framework. *Source* Authors' illustration

Figure 2 shows the disaster management structure at the national, regional, district, and chiefdom administrative levels.

The National Platform for Disaster Risk Reduction is the governing body in charge of the agency's management and supervision. During meetings, it reports to the chairman of the National Security Council. The platform comprises one administrative head from each of the relevant ministries, departments, and agencies of the central government (National Disaster Management Agency Act 2020).

The Agency Secretariat is in charge of providing technical and other assistance to the agency and the National Platform. The Director General is the administrative head of the Agency. The agency's functions are performed in the region, district, or chiefdom as directed by the national platform (National Disaster Management Agency Act 2020).

Management committees develop plans to prevent and mitigate the effects of disasters. At their respective levels, they prepare and implement disaster management plans. The committee comprises one administrative head from each of the relevant ministries, departments, and agencies at that level (National Disaster Management Agency Act 2020).

During a disaster, private sector organizations are expected to mobilize various institutional, personnel and financial resources to implement prompt and tangible actions necessary to address the consequences of disasters on their facilities or for which they are otherwise responsible (DMD and ONS 2006).

Local governments may coordinate with the central government, NGOs/INGOs such as the Sierra Leone Red Cross, Concern SL, and the respective community stakeholders in disaster management in their area of administration only (DMD and ONS 2006; Miles et al. 2021).

Community leaders are directly responsible for coordinating community resources to address the full spectrum of action to prevent, prepare for, respond to, and recover from disasters with external support. Civil society is a powerful pillar for transmitting disaster preparedness information, training, and volunteer activities to assist communities in becoming safer, stronger, and more resilient to catastrophes (DMD and ONS 2006).

Moreover, specialized Agencies of the United Nations in the country such as the UNDP and UNDRR, may support the central government, local governments and communities in disaster preparedness, mitigation, response and recovery (DMD and ONS 2006).

### Implementation drawbacks

**Fig. 3:**
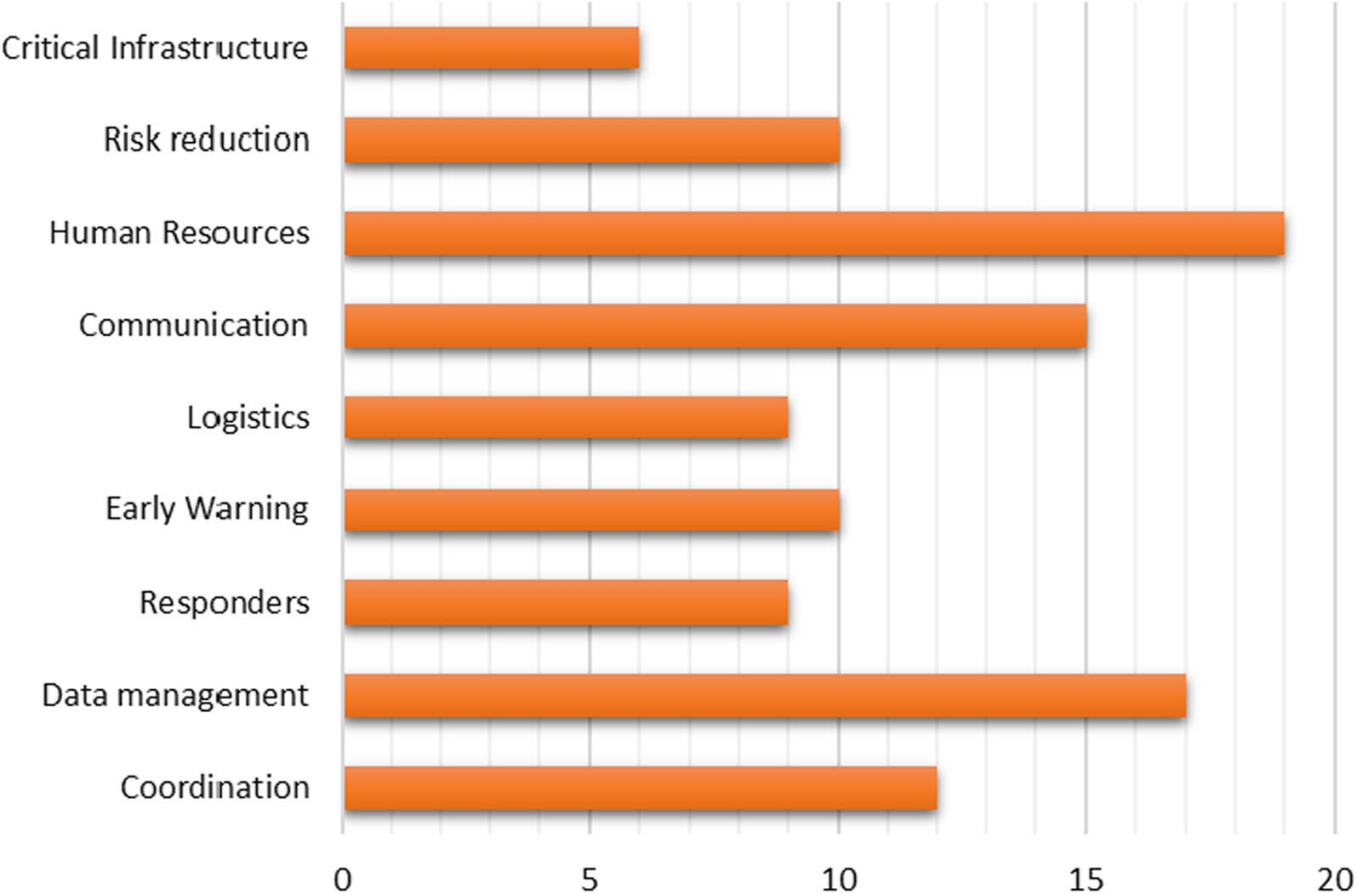
The disaster management drawbacks as drawn from the study. *Source* Authors

The issues hindering and being a drawback to the effective implementation of disaster management activities in the country were identified, examined and placed under human resources, communication, risk reduction, logistics and stock management, early warning, responders, data management, and coordination categories (Fig. 3). Financial constraints are not included in the classification since they are found in the literature as a widespread and underlying problem that affects how well workers are hired, trained, and involved in disaster management operations (DMD and ONS 2006; Government of Sierra Leone 2021; Miles et al. 2021).

#### Human resources

Most (17.8%) of the problems reported in the literature are related to human resource limitations. These factors include concerns such as the lack of professionals needed to meet the demands of the nation's disaster management efforts.

According to a study by Miles and others, the NDMA has a limited number of employed staff to cover all regions and roles, and only a few staff have specialised disaster management qualifications in the agency. The agency has a limited ability to manage simultaneous or cascading emergencies (Miles et al. 2021).

Apart from the inadequate staff in the NDMA, the Sierra Leone Meteorological Agency responsible for disaster warning has limited capacity in hydrological, marine, and agricultural meteorology. These issues have called for an urgent need to train people to repair and maintain stations rather than relying solely on international experts (Government of Sierra Leone 2021).

Furthermore, the Climate Change Secretariat of the Environmental Protection Agency has been highlighted as lacking the institutional strength to conduct a thorough vulnerability assessment, adaptation modelling, and adaptation planning within the country (Government of Sierra Leone 2021).

Furthermore, findings have also revealed that the Statistics Sierra Leone office cannot respond to the country's statistics and information demands in a coordinated and concerted manner with all stakeholders (Government of Sierra Leone 2021).

#### Data management

Data management drawbacks in disaster management accounted for 15.9% of all concerns raised in the literature. These concerns include issues with disaster-related data acquisition, storage, processing, retrieval, and use.

Research has revealed that the improper data collection process of affected persons during disasters is a leading cause of inaccuracies in disaster records. The lack of clarity surrounding revising data on affected people after initial collection and analysis is also a barrier to effective disaster data management (Miles et al. 2021).

Furthermore, the unavailability and inaccessibility of accurate data for disasters remain critical data management drawbacks (Miles et al. 2021; Osuteye et al. 2017). Osuteye and others found that there are not enough disaster databases to indicate the scope of losses in urban areas (Osuteye et al. 2017).

Most importantly, current disaster data collection is limited, does not cover all sectors, and is incompatible with current meteorological models. Also, the data's integrity and significance are sometimes questioned (Government of Sierra Leone 2021).

#### Communication

Communication issues that impede effective disaster management accounted for 14% of all concerns identified in the literature. The points raised here included inadequate and ineffective communication technologies, as well as expertise in coordinated disaster communication.

Previous research showed a lack of strategic disaster risk reduction communication and poorly prepared important messaging. The absence of robust and dependable internet access and an over-reliance on private mobile phones for disaster coordination are the primary causes of disaster communication failures (Miles et al. 2021).

Furthermore, Sawaneh highlighted the dissemination of false information about a disaster as a barrier to disaster management in his research and encouraged the government to take early action against this issue (Sawaneh 2020).

#### Coordination

Problems in coordinating disaster management activities accounted for 11.2% of the total issues in the literature.

According to research, the National Disaster Management Agency is seen by line ministries as a replacement rather than a coordinating partner. This issue, among others, has resulted in fewer official Disaster Management contact points in ministries (Miles et al. 2021).

Poor inter-pillar coordination at meetings, with more interest in reporting back activity rather than planning future aspirations, has also been noted as a barrier to effective disaster management operations (Miles et al. 2021).

Moreover, the government of Sierra Leone has recently noted that there is lack of effective collaboration among the national, regional and international institutions in managing disasters within the country (Government of Sierra Leone 2021).

#### Risk reduction

This category includes factors that raise individual and community risks of various disasters and hinder risk reduction. Only 9.3% of these concerns were discovered in the literature.

Research has identified illegal and substandard electrical installations as factors that increase the risk of electrically related fires and impede disaster risk reduction efforts. A notable instance of this was the Susan's Bay fire incident in November 2021, as electrical failure was one of the reported causes of the disaster (International Institute for Environment and Development 2021).

The unfocused disaster risk reduction messaging, particularly in educating communities before disasters, added to the poorly attended meetings on matters of disaster risk reduction by stakeholders and aided in increasing the risks and consequences of disasters in the country (Miles et al. 2021).

Another significant flaw identified by the literature in successful disaster risk reduction is the lack of updated hazard maps and risk registers for most districts and wards within the country (Miles et al. 2021).

Problems in maintaining quarantine integrity were identified during the Ebola Virus Disease outbreak. The desire to continue with traditional practices of caring for the sick increased the risk of transmission of the Ebola virus disease (Nally et al. 2022).

#### Early warning

From the literature sample, early warning drawbacks constitute 9.3% of the problems affecting successful disaster management plans and activities.

The country has been identified to have only basic early warning systems (Clark-ginsberg 2014). In addition, the staff's ability to quickly disseminate and interpret data to key policymakers is limited. As a consequence, there are problems for policymakers to fully comprehending early warning terminologies and interpretation (Government of Sierra Leone 2021; Miles et al. 2021).

#### Logistics and stock management

Inadequate logistic and stock management made up 8.4% of all disaster management problems in the literature. Among these concerns are the availability, accessibility, and sufficiency in good operating order of vehicles and other equipment required to respond to or avert a possible disaster.

The literature agrees that there is a limited stockpile of essential equipment required for disaster management.There are also challenges to maintaining up-to-date inventories of the available equipment at national and local levels (Government of Sierra Leone 2021; Miles et al. 2021). Also, overreliance on donor logistical capacities quickly overstretches them and may lead to donor fatigue (Miles et al. 2021).

#### Responders

As found in the literature, this category accounted for 8.4% of the disaster management problems in the country. It covers issues hindering emergency responders during operations, such as poor road networks, which delay response time, and a weak response system, which cannot coordinate adequate disaster responses on time.

The literature revealed a lack of maintenance budgets for first responders' equipment. There is a notable deficiency in the quality and quantity of operational equipment for the National Fire Force and the Sierra Leone Police, which are primary responders to disasters in Sierra Leone (Miles et al. 2021).

Moreover, the response system is mainly active in large-scale emergencies, leaving small-scale emergencies in danger of being sometimes overlooked or ineffectively responded to (Clark-ginsberg 2014).

Limited and, in many cases, no access to urban communities and informal settlements restricts responders' movement (Miles et al. 2021), This concern, combined with the inadequate know-how in first-aid and public hygiene for first responders, adds to the hindrance of effective disaster response (Shin et al. 2018).

#### Critical infrastructures

The inadequate infrastructure, such as hospitals, emergency evacuation centres and others, accounted for 5.6% of the total disaster management issues in the literature.

Miles and others pointed out that road networks have been receiving more investment in the country recently, but there remains a lack of coherent urban and strategic planning of road networks (Miles et al. 2021). Furthermore, the country lacks the medical facilities to supplement the surge capacity needed to avoid epidemics. Also, due to a lack of water supply, most healthcare facilities in Sierra Leone cannot manage their sewage systems properly (Shin et al. 2018).

### Authors' recommendations

**Fig. 4:**
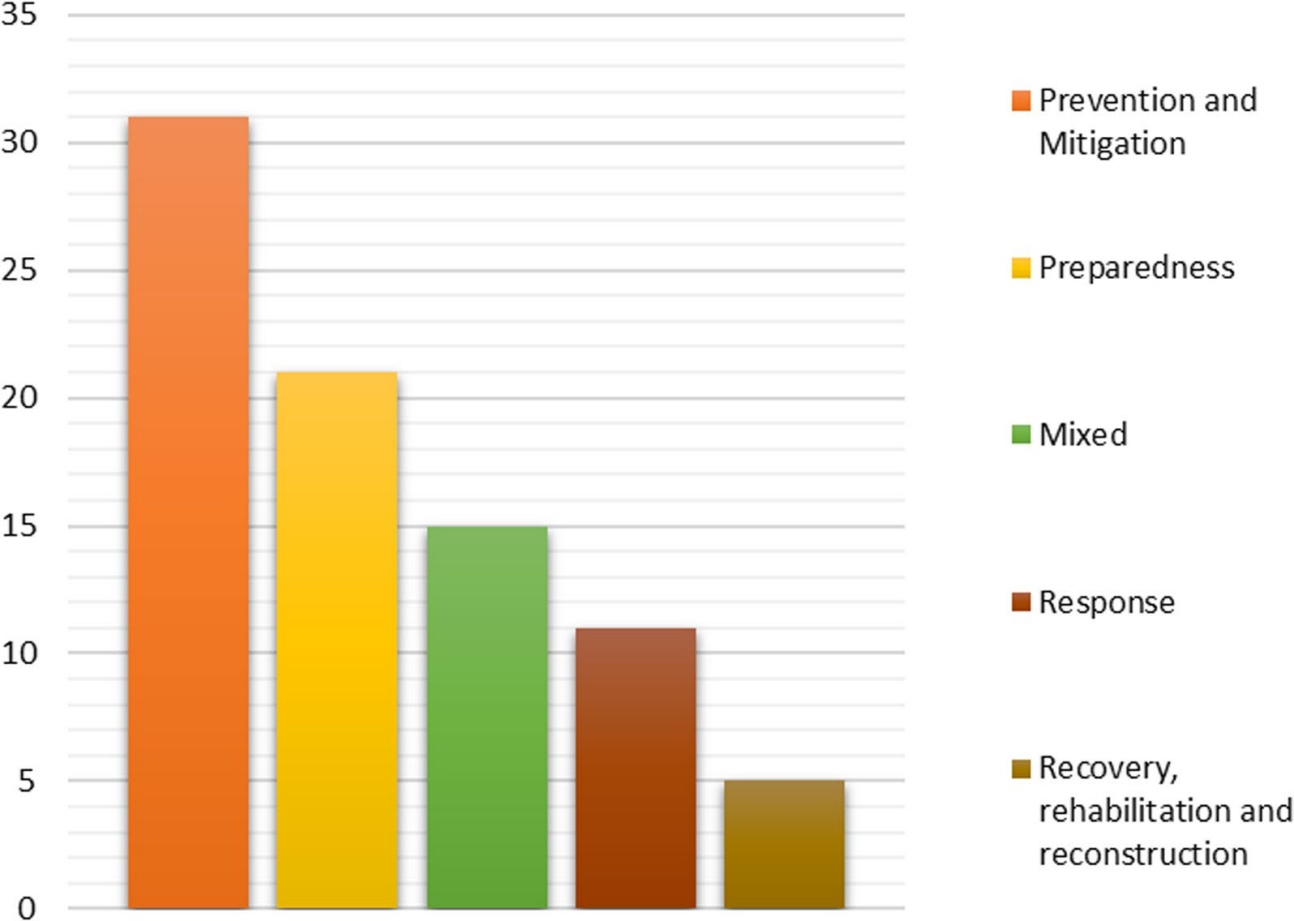
Authors' recommendations for disaster management. *Source* Authors

We analyze the authors' recommendations in the literature to comprehend their position as to what should be done in addressing disaster management concerns in the country. Eighty-two (82) recommendations were recorded from the sample. The recommendations were categorized following the disaster management phases: prevention, mitigation, preparedness, response, recovery/rehabilitation/reconstruction (See Fig. 4).

#### Prevention and mitigation

Recommendations about preventing and mitigating disasters were seen the most in the literature (37%). This finding shows the emphasis placed on the activities related to ensuring disaster does not happen or to lessen its impacts in case it does.

For instance, Auden and others recommended that signposts be placed in areas at risk of landslides in Freetown and that the Freetown City Council and the Ministry of Lands should increase transparency and accountability in giving the rights or prohibition to build to minimize building in areas prone to danger (Auden et al. 2017).

The Ministry of Lands Country Planning and the Environment (MLCPE) and the Freetown City Council (FCC) recommended that Gabions and traps be located and installed properly to restrict sediment transport, aid plant and soil stability, and, as a result, reduce run-off. They further stated that the existing urban planning and environmental protection rules be adequately enforced to prevent future encroachment and degradation of the urban and natural environments in and around the creeks (MLCPE and FCC 2014).

The City Council of Freetown and other responsible government institutions are further encouraged to relocate the King Tom, Granville Brook, and other informal disposal sites to more environmentally safe locations per modern standards (MLCPE and FCC 2014).

Runoffs have been caused by unlawful and indiscriminate trash dumping in streets and drainage systems. To curb these, taxes, fines, and other forms of punishment have been suggested (MLCPE and FCC 2014). These measures should be backed up by adequate storm drain, sewer management, and regular clearing (Miles et al. 2021).

A new Memorandum of Understanding and strategy for the National Disaster Management Agency that focuses on fostering greater cooperation with the country's electricity suppliers should be a top goal. This plan should concentrate on novel implementation measures to improve system robustness, resilience, and firefighting capabilities (Miles et al. 2021).

#### Preparedness

A total of 26% of the literature's recommendations are plans and actions that science suggests to be taken in preparing for disaster. These cover a variety of tasks, including early warning, education, and raising public awareness of disasters.

Previous work suggests that to prepare for water-related calamities, all government institutions concerned should provide education and training on the acquisition and use of life jackets, lifeboats, and life buoys for search and rescue, evacuation, and relief operations. The deployment of security forces in water catchment areas to prevent deforestation should also be strengthened (DMD and ONS 2006).

It has been advised that the National Disaster Management Agency lead a multi-agency communication policy and guidance to develop a culture of generating pre-disaster media messages and social media interaction formats across the NDM Agency, ministries, and local actors (Miles et al. 2021).

Miles and his colleagues suggested that a multi-agency and local community leader Early Warning System (EWS) technical dissemination training exercise be developed, emphasising establishing focal expert contact points with experience in analysing EWS-generated information and interpretations (Miles et al. 2021). They Furthermore stated that local stockpiling, physically secure storage, and warehouse management solutions must be prioritised locally (Miles et al. 2021).

Along with the above, Musoke and colleagues recommended that national health authorities do pre-disaster mapping of all partners participating in emergency response (Musoke et al. 2020).

Sawaneh and Turay further suggested that the government invest in science, engineering, and disaster research at universities to help them uncover innovations in disaster risk management and information technology (Sawaneh 2020; Turay 2022).

There is an agreement in the literature that to be adequately prepared for climate change uncertainties, all institutions' climate databases should be strengthened and kept up to date, including the provision of computer equipment and expert training in the entry and storage of climate-related data (Government of Sierra Leone 2021; Miles et al. 2021).

#### Response

Disaster response is the phase following the occurrence of an incident. 14% of all recommendations in the literature are for actions to take at this phase.

There is a consistency in researchers' emphasis on the need to strengthen operational response centres to match modern standards capable of handling rapid cases in situations like epidemics and mudslides (Musoke et al. 2020; Shin et al. 2018).

For instance, Musoke and his colleagues proposed that the National Public Health Emergency Operation Centre's activities be strengthened by ensuring that emergency response protocols are in place, are routinely updated, and that the duties of various sectors are well defined. They also recommended that government agencies in charge of the response keep a running list of survivors and their injuries for better documentation of the affected people (Musoke et al. 2020).

Incorrect information during a catastrophe can sabotage and complicate disaster response. To address this concern, Sawaneh proposed that those who spread fake news and misinformation be punished for setting a precedent (Sawaneh 2020).

Contentions between responders and the community often arise due to misunderstanding. In this regard, Shinn and others recommended that responders prioritise understanding the local communities' cultural norms and values to avoid conflicts of interest and attract collaborative efforts (Shin et al. 2018).

#### Recovery, rehabilitation and reconstruction

Recommendations about the appropriate actions to undertake during the recovery, rehabilitation, and reconstruction phase of a disaster in the country are the least (6%) in the literature.

The restoration of jobs and adequate housing to replace that which has been destroyed, as well as the restoration of the disaster area(s)' economic base through the National Commission for Social Action in collaboration with other stakeholders, are all recommendations to help rebuild better after a disaster (DMD and ONS 2006).

When residents in landslide-prone areas are forced to relocate, effective government response is essential to ensure that new destinations with adequate infrastructure and sound connections to the city are accessible (Auden et al. 2017).

#### Mixed

General recommendations about solving different disaster issues that cannot be ideally placed in any of the disaster management phases fall under this category; they account for 17% of the overall recommendations in the literature.

For instance, Clark-ginsberg recommended developing a disaster risk reduction program that focuses on governance, physical mitigation, readiness, and recovery, with community disaster management emergency response teams serving as specific partners (Clark-ginsberg 2014).

Miles suggested revising the "Criteria for Declarations of Emergencies and Thresholds for NDMA Interventions." He further advised launching a new planning project focused on funding smoother transitions between response and recovery. Those moves will assist in driving future contingency financing arrangements and priorities (Miles et al. 2021).

Musoke proposed that District Disaster Management Committees be described and duties assigned to coordinate district emergency response operations to better prepare and respond at the district level (Musoke et al. 2020).

#### Research gaps

The following are ideas or concepts that have not been investigated at all. They include problems not solved or questions not answered by the existing research in this field. As a result, the situation demands immediate action for the country to manage disasters effectively.

Nally and others call for the examination of methods that involve all stakeholders at the start of any disaster response in the country (Nally et al. 2022). The study of barriers to early warning system information is required (Clark-ginsberg 2014).

To improve knowledge in this field, a meta-analysis is needed to compare the effectiveness of international non-governmental organisations' (INGOs) response activities in different contexts and response periods for different types of diseases (Shin et al. 2018). Also, a meta-analysis of studies to understand extreme poverty in Freetown slum areas and the impacts of small-scale emergencies is required (Clark-ginsberg 2014).

More research on community-based solutions during an emergency response is required (Nally et al. 2022). It is also necessary to further assess the capacity of community disaster management and emergency response teams to improve their abilities (Clark-ginsberg 2014).

Clark-Ginsberg has further requested the assessment of slums and micro-slums, focusing on changing vulnerability profiles. He also pointed out that a review of the causes of corruption in Sierra Leone is necessary, as corruption has been identified as one of the disaster management obstacles (Clark-ginsberg 2014).

A comprehensive assessment of droughts is required, which includes a realistic representation of the water available in soils, drought propagation, feedback from vegetation cover, and human influence during these events (Henchiri et al. 2021).

Additional geotechnical drilling and investigation are required to provide deep-seated structural information, knowledge of secondary minerals present in joints, and characterisation of soil material in the hills (Lahai et al. 2021).

Moreover, more must be done to integrate health and disaster research and sectors to address risks better (Osuteye et al. 2017).

To analyse and identify training, technical, and financial support, a thorough assessment of climate service capacities, including all climate-sensitive institutions, is required (Government of Sierra Leone 2021).

There is a scarcity of data on local and national vulnerabilities, impacts, and risks. A comprehensive exposure and risk assessment of all sectors and regions must be conducted and publicised (Government of Sierra Leone 2021).

To detect emerging outbreaks, studies characterised by the epidemiological profiles of Lassa Fever should remain a priority at the local level. Also important in the fight against Lassa fever are monitoring, evaluation, diagnostic testing strategies, and facilitating Lassa Fever vaccine studies (Shaffer et al. 2021).

### The advances and prospects

The following is the presentation of the advances and prospects for disaster management in Sierra Leone according to the literature.

The establishment of the Disater Management Department in 2004 was a step in the right direction for a country that did not have an institution dedicated to coordinating and managing disasters (DMD and ONS 2006).

Following that, the government of Sierra Leone (2006) developed a comprehensive disaster management policy that clearly states the aims, objectives, strategies, roles and responsibilities of coordinating institutions and implementing agencies (Sierra Leone Disaster Management Policy 2006).

The 2007 National Adaptation Program of Action, based on the goals and objectives of the Poverty Reduction Strategy Paper and the Millennium Development Goals, linked adaptation to national development planning and international development goals (Government of Sierra Leone 2021).

Thereafter, in response to the World Climate Conference in 2009, the National Framework for Climate Services was established, with the goal of strengthening the design, delivery, and implementation of climate services across sectors and localities (Government of Sierra Leone 2021).

In 2010, the strategy for the Development of a Climate Change Abatement Economy was developed, focusing on opportunities for earning forest carbon credits through implementing the REDD/REDD + program (Government of Sierra Leone 2021).

A participatory and iterative process was used to develop the National Climate Change Policy Framework in 2012 to improve the national capacity to adapt to climate change. In 2015, the National Climate Change Strategy and Action Plan were developed, advancing the country's climate policy to include adaptation actions in agriculture, adaptation to sea-level rise, tourism, fisheries, forestry, health, and water resources (Government of Sierra Leone 2021).

Furthermore, the Environment Protection Agency developed the Integrated Coastal Zone Management Plan 2016–2020 in 2015 for effective coastal management (Government of Sierra Leone 2021).

Establishing the Sierra Leone Meteorological Agency in 2017 with the mandate to manage the early warning system is a huge benefit to the country's effective disaster management (National Disaster Management Agency Act 2020).

The national adaptation planning process in 2018, and the framework development in 2019, demonstrated the country's intention to reduce vulnerability through adaptation mechanisms (Government of Sierra Leone 2021).

Through the 2018 National Land Degradation Neutrality target-setting process, Sierra Leone's Technical Working Group identified and established hotspots of degraded lands. The National Drought Management Plan for Sierra Leone was created the same year as a contingency plan for Sierra Leone (Government of Sierra Leone 2021).

Sierra Leone has completed three national communications to the UNFCCC to meet its global commitments. The first national communication was completed in 2007, followed by the second in 2012 and the third in 2018 (Government of Sierra Leone 2021).

The transition from the Disaster Management Department to a dedicated National Disaster Management Agency in 2020 represents a significant step forward in the country's disaster management (National Disaster Management Agency Act 2020; Miles et al. 2021).

Since 2020, local disaster management capacity has been improved, allowing the Freetown City Council to respond to 14 fire incidents in 2021, among other things. The Sierra Leone Meteorological Agency was said to be reinforced by UN Development Programs with software packages in 2021; if used appropriately, this support will improve the agency's capacity (Miles et al. 2021).

The implementation of After-Action Reviews (AARs), and the coordination of recovery planning between the central and regional ministries, departments, and agencies, as well as the formation of a Technical Working Group (TWG) for disaster management are steps forward, and a road map for effective disaster management (Miles et al. 2021).

The National Determined Contribution was updated in 2021 to increase adaptive capacity, strengthen resilience, and cut vulnerability in half by 2030 (Government of Sierra Leone 2021).

## Conclusion and recommendations

This paper has provided essential information necessary to understand the contemporary context of disasters, their management, implementation drawbacks, research gaps, the advances made so far, and the prospects for disaster management in Sierra Leone. The findings contained in this paper, if taken into consideration, will provide ways to improve the country's disaster management.

A critical review of the literature revealed that newer studies do not provide adequate follow-up on the gaps and limitations of previous related works on this subject, resulting in some disconnections in the literature.

Financial constraints have been a primary cause and underlying hindrance to disaster management. Apart from that, the inadequate specialised and ineffective disaster management staff, lapses in data management and inefficiencies in communication among the relevant institutions and communities, as well as within the institutions themselves, are the most pressing considerations for improving disaster management in the country.

Fewer disaster events were published compared to those observed being posted on Twitter, Facebook, and WhatsApp. Future research is required to assess the relevance and influence of the named social media tools in informing the science and practice of disaster management in Sierra Leone and how they can influence disaster communication at the community and institutional levels in Sierra Leone.

Simulating and modelling studies for landslides, mudflows, rockfalls, flooding, and outbreaks comparable to Ebola and Corona Virus diseases at the local level are encouraged to provide knowledge for mitigation and preparedness for probable future occurrences.

The role of volunteers in times of disasters and emergencies often goes unnoticed, unrecognized, or unrewarded. This concern may lead to low morale and motivation. Considering this, the authors agree with previous researchers and emphasize the importance of the Sierra Leone government incorporating community-based disaster volunteers into the national disaster management structure of the agency.

This paper recommends that the National Disaster Management Agency work with the local communities and other partners to develop an updated national disaster risk and vulnerability profile using primary data and GIS techniques. Communities should be demarcated into zones based on the magnitude of risk and vulnerability to the particular types of hazards to which they are exposed. Following these activities, there should be mass sensitization at the individual community levels to inform them about their exposure and vulnerability to the relevant hazards.

Prevention, mitigation, and preparedness received the most attention, while recovery, rehabilitation, and reconstruction received the least attention in the disaster management phases of Sierra Leone. This revelation is understood because effective prevention, mitigation, and preparedness will leave little room for a disaster to strike and the need to heal from it. However, in light of current climate change uncertainty, the authors recommend that contingency plans for economic recovery and the relocation of individuals in at-risk areas be strengthened. The contingency plans should be flexible to adapt to climate change uncertainties and be subjected to a periodical review by the relevant ministries, departments, agencies and their partner institutions.

The paper further recommended that the responsible institutions of the government of Sierra Leone launch an investigation to comprehensively understand the welfare and adaptation of relocated people in the country over time. Such an investigation will reveal essential information to learn from and improve existing resettlement homes and plans.

Disaster response requires adequate vehicles and other response equipment. As such, the prompt repair of vehicles, motorcycles, and fire response equipment must be given premium consideration to reduce the use of the limited available funds to purchase new ones or wait for donors to support such purchases.

Additionally, the agency should strengthen the disaster reporting channels at the community level and, where necessary, provide training for all community members to enhance their emergency preparedness and response actions. Moreover, an effective community feedback mechanism must be created to share experiences, effects, strategies, practices, and lessons learnt among communities.

The agency and its partners should embark on adequate nationwide sensitization, starting at the community level, on the appropriate use of emergency numbers (such as the health and fire emergency hotlines 117, 999, and police 019). Following this process, the agency should strengthen its intelligence systems to track and differentiate false alarms or prank calls and levy appropriate penalties where necessary. These measures will improve the reporting of incidents, reduce the misuse of emergency hotlines and facilitate prompt response.

The authors also recommend that the ministry of finance support the agency and other institutions involved in disaster management by facilitating the approval of appropriate disaster management projects. Similarly, the agency’s human resources department should be financially supported by the government of Sierra Leone and international partners to employ the necessary quantity and quality of staff required to implement critical nationwide disaster management plans.

In addition, all the operational staff of the agency should routinely be exposed to international tabletop exercises, simulated disaster response training, and seminars to help improve their capacities and international experience.

Disaster management modules and subjects in higher learning institutions in the country should not only be limited to seminars and teaching. Students should also be encouraged to be involved in disaster management research and practical aspects with supervision from senior agency staff.

Finally, the authors recommend that disaster management initiatives consider gender equity, situations of the disabled, and other vulnerable and marginalized groups in society at all levels of planning and implementation of activities.

## Data Availability

Not applicable.
